# Biotic stress-induced changes in root exudation confer plant stress tolerance by altering rhizospheric microbial community

**DOI:** 10.3389/fpls.2023.1132824

**Published:** 2023-03-10

**Authors:** Indrani Sharma, Sampurna Kashyap, Niraj Agarwala

**Affiliations:** Department of Botany, Gauhati University, Gopinath Bordoloi Nagar, Jalukbari, Guwahati, Assam, India

**Keywords:** biotic factors, rhizospheric microbial community, root exudates, stress tolerance, plant microbiome

## Abstract

Every organism on the earth maintains some kind of interaction with its neighbours. As plants are sessile, they sense the varied above-ground and below-ground environmental stimuli and decipher these dialogues to the below-ground microbes and neighbouring plants *via* root exudates as chemical signals resulting in the modulation of the rhizospheric microbial community. The composition of root exudates depends upon the host genotype, environmental cues, and interaction of plants with other biotic factors. Crosstalk of plants with biotic agents such as herbivores, microbes, and neighbouring plants can change host plant root exudate composition, which may permit either positive or negative interactions to generate a battlefield in the rhizosphere. Compatible microbes utilize the plant carbon sources as their organic nutrients and show robust co-evolutionary changes in changing circumstances. In this review, we have mainly focused on the different biotic factors responsible for the synthesis of alternative root exudate composition leading to the modulation of rhizosphere microbiota. Understanding the stress-induced root exudate composition and resulting change in microbial community can help us to devise strategies in engineering plant microbiomes to enhance plant adaptive capabilities in a stressful environment.

## Introduction

Being sessile in nature, plants need to withstand changing environmental conditions to ensure their survival. Adapting to fluctuating conditions, plants require the adoption of various sophisticated mechanisms by molecular, physiological, or morphological changes. Emerging research in plant science is very less concerned with the rhizospheric microbial community shaped by the biochemical compounds present in the root exudates delivering adaptive capabilities during stressful environmental conditions. However, it is very interesting that plants themselves invest up to 40% of their photosynthetically assimilated carbon as exudates into the rhizosphere primarily composed of a wide range of compounds, e.g., a blend of amino acids, compound sugars, and organic acids (Canarini et al., 2019; Bhutia et al., 2023). Plants are endowed with the capability to alter plant rhizospheric microbes by natural means such as root exudation, which is crucial for plant growth and development. The root exudation pattern of crop wild relatives is attracting much attention nowadays, as root exudates modulating the rhizosphere microbiome might have the potential to substitute the use of chemicals in agricultural fields (Preece and Peñuelas, 2020). Recently, scientists have developed the concept of entropy-based engineering of the rhizosphere, which targets more production using less input (Zhang et al., 2022). With the advances in understanding the role of root exudates in modulating the rhizospheric microbial community, it can be correlated that under any environmental constraints, root exudation pattern changes, resulting in the alteration of the root-associated microbiome. Understanding the role of root exudates is not possible without discussing various environmental conditions that can play pivotal roles in shaping the rhizospheric microbial community. Changing climatic conditions exert pressure on the plants, which are ultimately executed by the roots in shaping the root system architecture (RSA), depending upon the plant developmental stages and genotypes (Dabhi et al., 2021; Sarita et al., 2022). The RSA helps in the better adaptation of the plants through enhanced nutrient acquisition and by modulating the rhizospheric microbial community. In addition to RSA, various edaphic factors like soil pH, soil temperature, aeration, and physicochemical characteristics have a great influence on determining the microbial community of the rhizosphere (Ling et al., 2022). Root exudation pattern and rhizosphere microbiome structure also change in accordance with the type of soil where the plant grows (İnceoğlu et al., 2012).

In addition to the above-listed edaphic conditions determining the composition and quantity of root exudates, it also firmly depends upon specific plant species or host genotype, prevailing environmental conditions, and the stress status of the plant. The root exudates act as a signalling molecule and help in recruiting beneficial root-associated mutualists, like *Pseudomonas*, *Bacillus*, *Trichoderma*, and mycorrhizal species (Pieterse et al., 2014), and eradicate harmful microbes in the rhizosphere, resulting in shifting the microbial community composition. Plant-associated rhizospheric microbes assist their host in a variety of activities, ranging from nutrient acquisition to defence against herbivores (Malacrinò et al., 2021), by activating induced systemic resistance (ISR) where root-specific transcription factor MYB72, phytohormones like salicylic acid (SA), volatile organic compounds (VOCs), and defence regulatory proteins play a critical role (Pineda et al., 2010; Pieterse et al., 2014). Plants respond robustly against insects and pathogens attacks by priming their leaf tissues with the help of ISR rather than directly activating the defence-related genes (Pineda et al., 2010). Traditionally, the main objectives underlying plant growth-promoting microorganism research include plant growth stimulation and yield enhancement. Understanding the role of root exudation in altering microbial community dynamics has recently emerged and attracted much attention. Further, the rhizospheric microbes and their molecular mechanisms involved in plant–plant interaction for establishing social networking systems have been studied in detail. The mycorrhizal fungal associations and the parasitic plants play significant roles in generating wired social networking systems. In contrast, root exudates and VOCs establish a wireless social networking system among the neighbouring plants (Park and Ryu, 2021; Sharifi and Ryu, 2021).

This review highlights various biotic stress-inducing factors that result in the amendment of rhizospheric microbial community composition. Firstly, we gave an overview of the microbes generally found in the rhizosphere followed by the role of different environmental factors (major biotic factors) influencing the host root exudates composition in the recruitment of either beneficial or pathogenic microbes. Secondly, we discussed the importance of root exudates in influencing the community structure of plant microbiomes. Further, based on various studies, we have tried to decipher the importance of positive and negative below-ground interactions in influencing plant resilience against varied environmental circumstances.

## The rhizosphere and its microbiome

The soil is an overwhelming reservoir of microbial diversity. It has been estimated that 1 g of soil can carry billions of bacterial cells belonging to thousands of different taxa having crucial functions in plants and the environment. These microbial complexes establish an association between plant and rhizosphere and are also considered as the plant’s second genome (Berendsen et al., 2012). Hiltner in 1904 defined rhizosphere as the soil region that is directly associated with plant roots. With a deeper understanding of the rhizosphere as the hotspot of soil biogeochemical cycles, the definition of rhizosphere has been extended. Based on the paradigm of various soluble compounds and gaseous molecules, a new concept of rhizosphere has been developed, which defines it as the portion of soil involving the root system, up to which gaseous exchange by means of diffusion can take place (de la Porte et al., 2020). Contemporary investigations in understanding the rhizosphere microbiome have revealed that the host plant’s genetic makeup is also involved in the alterations of the rhizomicrobiota. The rhizosphere microbiome is very important for the fitness of the host, and the manipulation of the rhizosphere microbiota is presumed to provide a solution for climate-resilient crop production (Pieterse et al., 2016). A competitive battle in the rhizosphere is going on among different types of microorganisms competing for plant-derived nutrients, resulting in their interactions with the roots (Li et al. 2021b). For instance, arbuscular mycorrhizal fungi (AMF) are reported to colonize approximately 71% of terrestrial plants exhibiting complex interaction patterns in various plants except for plants belonging to Brassicaceae, Chenopodiaceae, Polygonaceae, Amaranthaceae, and Caryophyllaceae (Wang and Qiu, 2006; Fernández et al., 2019). Complex chemical signalling between the host plant and AMF results in enhanced plant nutrient acquisition and protection of the plants from pathogen infection (Dowarah et al., 2021). Sensing the varied environmental stimuli, the microbiome structure of the soil changes, which is further interlinked with the nature of the soil and the plant species involved in the recruitment of microbes (Garbeva et al., 2008). İnceoğlu et al. (2012), from their investigation, reported that the type of soil functions as a determining factor in structuring the rhizospheric microbial community. Based on the rhizospheric effect, they concluded that potato cultivars grown on different soil types recruited diverse communities of microbes with diverse functional abilities. Five types of potato cultivars were collected from loamy and sandy soil regions and found that bacterial communities in the loamy soil rhizosphere show more affinity towards carbohydrates and amino acids, while communities reported from the sandy soil rhizosphere have shown a higher affinity towards polymers, alcohols, and nucleotide-based carbon sources, which were secreted out by the roots as exudates. Despite these natural signalling cues, a new concept of “entropy” has been introduced in the area of agricultural research, which tries to emphasize that the lower the entropy, the more ordered the rhizospheric interactions, which can ensure proper utilization of energy and resources by the plant (Zhang et al., 2022).

Plenty of research on soil microbial communities revealed that soil around the root region has a high density of microbes such as Actinomycetes, bacteria, and fungi, with higher activity rather than the bulk soil, a phenomenon known as the “rhizosphere effect” (Philippot et al., 2013; Bakker et al., 2020). However, as compared to bulk soil, the rhizosphere harbours a less diverse group of microbes (Philippot et al., 2013; Bakker et al., 2020). Depending upon environmental stimuli, the rhizosphere acquires selective microbes from the bulk soil. In the rhizosphere, the most prominently reported bacterial groups are the Acidobacteria, Bacteroidetes, Firmicutes, Planctomycetes, and Proteobacteria (Uroz et al., 2010; Bulgarelli et al., 2012; Dastogeer et al., 2020). Moreover, some bacterial communities like Actinobacteria and Xanthomonadaceae are scarcely present in the rhizosphere as compared to the bulk soil (Bulgarelli et al., 2012; Dastogeer et al., 2020). It has also been reported that the rhizosphere is predominantly colonized by mycorrhizal fungi that are estimated to be associated with approximately 80% of all land plants providing N and P for proper growth and development (van der Heijden et al., 2015; Weng et al., 2022). This symbiotic interaction between the mycorrhizae and the roots of higher plants has great significance in shaping the terrestrial ecosystem, as it can regulate the carbon and other nutrient cycles of the earth.

## Principal engineers of the rhizosphere microbiome

Plant rhizosphere microbiome is a result of interaction among plants, microbes, and associated environmental factors in either biotic or abiotic form or in alliance forming a tritrophic interaction (Badri et al., 2009). The plant species, its genotype, distinct tissue or organ, age and developmental state, ecosystem canopy type, immunity, etc., are some of the factors associated with the host plant, which can determine the rhizospheric microbial community (Dastogeer et al., 2020). Together, several plant-derived compounds like different alkaloids, phenolics, and terpenoids also have an important role in shaping the rhizosphere microbiome. Some of these plant-derived compounds are specific for some plants; e.g., glucosinolates are found predominantly in the Brassicaceae group (Dastogeer et al., 2020), and aglycone benzoxazinoids (BX) are produced by maize, which induces jasmonic acid-dependent resistance against herbivores (Hu et al., 2018).

In addition to the above-ground environmental factors and plant genotype, the rhizospheric microbial community composition relies also upon the soil texture and below-ground abiotic factors such as soil temperature, pH, and CO_2_ concentration. Fierer et al. (2007) investigated and found that carbon availability in the soil can play a role in the regulation of certain bacterial community structures involving Acidobacteria, Bacteroidetes, and Betaproteobacteria. However, Lauber et al. (2009), in their study, revealed that soil pH has a remarkable effect on the regulation of soil microbiome structure. On the contrary, the temperature was found to modulate plant root interactions with rhizospheric microbes, where the rhizosphere was found to be more responsive to changing temporal conditions than the phyllosphere (Luo et al., 2020); e.g., roots colonized by mycorrhizal fungi shows greater resilience in temperature stressed condition, playing a key role in plant adaptation to high temperature (Usman et al., 2021).

In contrast to various environmental factors, biotic components such as pathogens, parasites, or mutualistic plant microbes, present at the phyllosphere or rhizosphere, perform a crucial role in shaping the rhizospheric microbial community by influencing the survival of plants during or post-stress conditions. Among various biotic factors, AMF actively takes part in shaping the rhizomicrobiota (Dowarah et al., 2021). AMF association with the plant root is a very complex process that requires coordination between plant and fungal signalling molecules. Roots having AMF associations are protected against pathogens as AMF helps in nutrient acquisition and repair of pathogen-damaged cells (Dowarah et al., 2021). Here, the root architecture plays an important role, as it was reported that AMF benefit the tap root system in a better way as compared to the fibrous root system (Yang et al., 2015). Frew and Price (2019) reported that under elevated CO_2_ levels, both C3 and C4 plants produce a high amount of photosynthetically fixed C compounds. C4 plants allocate their excessive photosynthates in the rhizosphere with the help of root exudation, which leads to better AMF colonization, finally resulting in enhanced nutrient acquisition than that of C3 plants. Data on the underlying mechanism(s) involved in rhizosphere microbiome amendment under the influence of various stress conditions are limited. In this review, we have tried to highlight the role of biotic stresses in altering root exudate secretion patterns and also how the change in root exudate composition can influence the recruitment of the rhizospheric microbes during stress conditions.

## Mechanism of root exudation

Root exudation is the key mechanism by which plants maintain a homeostatic interaction with their below-ground environment. Plants exude various primary and secondary metabolites as root exudates, ultimately shaping the community structure of rhizosphere-dwelling microbes. The exact mechanism of root exudation is not yet clear, but to allocate the photosynthetically fixed C from the aerial plant parts to the soil, a mechanism similar to Munch’s hypothesis (Münch, 1930) is used by the plants, where diffusion plays a critical role (Canarini et al., 2019). According to Munch’s hypothesis, a concentration gradient between the source and sink organs, i.e., phloem and root tip generate turgor differences, eventually resulting in the translocation of metabolites from the phloem to the root tip and then to the soil (De Schepper et al., 2013; Ross-Elliott et al., 2017). Exudation of various metabolites from the root cells to the soil might involve facilitated diffusion, where concentration gradients act as a central force and specific transmembrane proteins help in the efflux of such molecules (Sasse et al., 2018). Specialised efflux carriers and channel proteins facilitate the secretion of primary metabolites like amino acids, organic acids, and sugars into the rhizosphere. For example, for amino acids, UMAMIT (usually multiple acids move in and out transporter), CAT (cationic amino acid transporter), and GDU (glutamine dumper) transporters, for organic acids, ALMT/malate (aluminium-activated malate transporter) and MATE/citrate transporters (multidrug and toxic compound extrusion), and sugar molecules, a family of transporters known as the SWEET transporter family are used by the plants (Canarini et al., 2019). Like primary metabolites, secondary metabolites also constitute a part of root exudates. Various secondary metabolites like phenolics, alkaloids, and terpenoids are transported into the rhizosphere with the help of ATP-binding cassette (ABC) transporters (Badri et al., 2009), whereas most of the primary metabolite transporters use passive transport as a mechanism of exudation, except MATE/citrate transporter having H^+^ coupled antiport activity (Meyer et al., 2010). A balance in the concentration gradient across the external and internal environment is maintained by the rhizospheric microbes as well as by the plants themselves. The exudates secreted out are consumed by various rhizospheric microbes, and a low concentration level is maintained in the external environment, enhancing root exudation (Canarini et al., 2019). With the help of phloem loading and transport (source activity), phloem unloading (sink activity), regulation of the expression of genes, or post-translational modifications of efflux carriers, plants might control the process of root exudation. Overall, the plant source-sink dynamics fundamentally regulate the allocation of photosynthates into the soil (Farrar and Jones, 2000; Savage et al., 2016). Moreover, plants modify their RSA depending on the concentration of primary metabolites at the root region, thus controlling the exudation process (Canarini et al., 2019; Bhutia et al., 2023). Modification in the efflux or influx of metabolites at the root region critically shapes the RSA where amino acids have a cascading influence in shaping the RSA. The expression of NRT2.1 transporter genes is mainly regulated by the amino acids, indicating the plant-soil N status (Nazoa et al., 2003). Glutamate is an important amino acid that functions similarly to the phytohormone auxin. A rapid increase in cytosolic Ca^2+^ and depolarization of the membrane is induced by glutamate, where an electrical signal similar to the synapsis of mammals is generated (Baluška and Mancuso, 2013; Ni et al., 2016). Additionally, a receptor family, known as the glutamate receptor-like (GLR) receptors, functions like amino-acid-gated Ca^2+^ ion channels and acts as a signal to convey the plant N status to the root (Brian and Lea, 2007; Vincill et al., 2012), thus shaping the RSA. The change in RSA, impacts the root exudation pattern by changing the composition of root exudates under various environmental conditions.

Since the exudation of various metabolites involves specialised carriers and takes place through facilitated diffusion, the molecular mechanisms involved in such processes may indirectly govern the mechanisms of root exudation. Further, studies on genes involved in shaping the RSA of plants might help in the detailed understanding of the root exudation mechanism.

## Diverse role of root exudates and biotic factors in shaping the rhizosphere microbiome

The ability of plants to adapt and survive in an environment primarily depends upon their capacity to acquire resources and resist external perturbations, impacting growth and productivity. In non-homogeneous soil conditions, the plant roots perform a crucial function in the absorption of mineral and nutrient resources from the soil, either directly or in association with rhizospheric microbes. These microbes are presumed to be attracted by plant-specific root exudates under normal or stressful circumstances. Thus, nutrient supply effect root exudation mostly from the root apices, ultimately altering the nutrient dynamics and the soil microbiome structure (Paterson et al., 2006).

**Figure 1:**
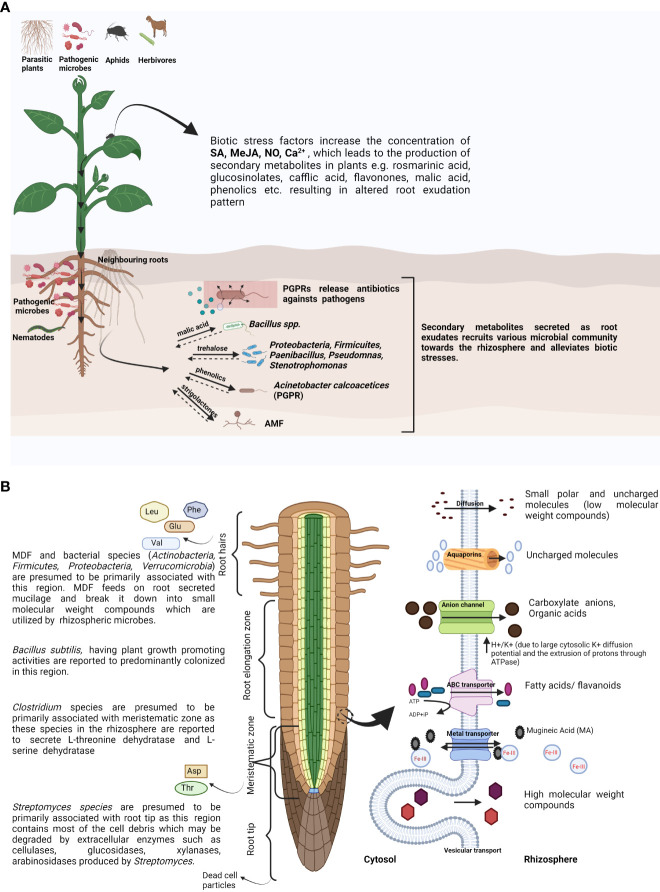
**(A)** Schematic representation showing the role of root exudates in altering rhizospheric microbial community composition sensing above-ground and below-ground biotic stresses. **(B)** Schematic representation of distinct root zones secreting varied blends of compounds along with the possible secretion processes across the plasma membrane into the rhizosphere. SA, salicylic acid; MeJA, methyl jasmonate; NO, nitric oxide; AMF, arbuscular mycorrhizal fungi; VOCs, volatile organic compounds; PGPR, plant growth-promoting rhizobacteria; Leu, leucine; Phe, phenylalanine; Glu, glutamic acid; Val, valine; Asp, aspartate; Thr, threonine; ABC transporter, ATP-binding cassette transporter. Created with BioRender.com.

The quality and quantity of various root exudates with different compositions secreted from different locations of the root are mainly determined by root branching and RSA (Badri and Vivanco, 2009; Canarini et al., 2019). The root meristematic and elongation zone present above the root tip secrete amino acids like asparagine and threonine, while other amino acids like glutamic acid, valine, leucine, and phenylalanine are secreted from the root hairs, along with the whole root exuding aspartic acid (**Figure 1B**) (Haldar and Sengupta, 2015). Several discrete blends of phytochemicals from various plant species are also released from different parts of the root like the mature root regions and root cap and also from root hairs (Nguyen, 2009).

Exudation from the roots contributes to both positive and negative associations in the below-ground environment. Positive root–microbe associations involve mutualistic interactions between root and beneficial microorganisms like plant growth-promoting rhizobacteria (PGPR) and mycorrhizal fungi, while negative associations involve the interaction of roots with harmful organisms like pathogens, invertebrate herbivores, and parasitic plants (Badri et al., 2009). Plants produce diverse arrays of secondary metabolites such as phenolic compounds, alkaloids, and terpenoids, some of which are genus/species-specific (Bednarek and Osbourn, 2009; Dastogeer et al., 2020). For example, barley plants infected with *Fusarium graminearum* release exudates having a high concentration of phenolic compounds, which alters the rhizospheric microbial community to enhance the plants’ defence against *F. graminearum* (Phour et al., 2020; Afridi et al., 2022).

Microbial degradation of leaf litter and dead plant substances in the rhizosphere serve as key resources for plant growth and also participate in carbon storage in the ecosystem by releasing nutrients as a decomposition product (Schneider et al., 2012). Studies suggest that mucilage-degrading fungi (MDF) and other microbes actively work upon mucilage or root exudates and break them into small molecular weight compounds, which act as signalling molecules for the recruitment of various PGPR. *Artemisia sphaerocephala*, a remarkable plant of Asian cold deserts, was used in a study where mucilage-degrading microbes were isolated from the root–soil junction. From the study, it becomes evident that the MDF associated with the roots of *A. sphaerocephala* recruit bacterial groups like Actinobacteria, Firmicutes, Proteobacteria, and Verrucomicrobia towards the roots and regulate the rhizosphere microbiota (Hu et al., 2021). Further, microfluidics-based investigation for real-time study of plant–microbe interaction revealed that *Bacillus subtilis* exhibits chemotactic behaviour towards the root elongation zone, which might be due to the secretion of root exudates (Massalha et al., 2017; Sasse et al., 2018). In addition to that, *Escherichia coli* cells from the rhizosphere were excluded after the colonization of *B. subtilis* at the rhizospheric region of *Arabidopsis thaliana* (Massalha et al., 2017). Microbes belonging to the group *Clostridium* are presumed to be associated with the root meristematic region as they produce l-threonine dehydratase and l-serine dehydratase enzymes, which may catalyze dehydration and isomerization of root-secreted threonine and serine, respectively (Hofmeister et al., 1993), and might alter the root exudation pattern of the host plant. *Clostridium* spp. are also involved in the degradation of renewable complex compounds like lignocellulose and make it utilizable for the plants as a resource, ultimately maximizing the C substrate exploitation and yield of the plants (**Figure 1B**) (Du et al., 2020). The root tip actively associates with a number of bacterial groups and withstands numerous obstacles due to which the cells shed off and reform in a continual process. These damaged cell parts contain lignin, cellulose, and various organic compounds and are presumed to be degraded by the *Streptomyces* species, as it contains a large amount of extracellular enzymes such as cellulase, glucosidase, xylanase, and lignocellulase (Schrempf et al., 2007; Chater et al., 2010).

Since the plants encounter a range of above-ground and below-ground constraints in their lives, to cope with those factors, the plants secrete different blends of chemical compounds in the form of root exudates, which varies in different plants with respect to their age. Nevertheless, it is very difficult to understand the factors responsible for variation in root exudation, as it is an emerging area in plant biology, and more precise research needs to be performed in the future.

### Belowground chemical signalling molecule acting as a messenger between plant and rhizosphere microbiome

Plants, being solitary in nature, have developed sophisticated defence mechanisms to resist the attack of pathogenic microbes as well as herbivorous organisms. It is evident that plants alter their primary and secondary metabolic pathways to withstand biotic stress, but there is very little knowledge that the roots have a key role in the regulation of those pathways for better adaption of the plants. Ongoing research on root exudates revealed that the roots take part in the synthesis and secretion of phytotoxins, helping the plants to sense their surroundings, leading to the activation of defence signals (Erb et al., 2009). Further, secretions from neighbouring plants have effects on one another and can induce changes in root exudate compositions (Ulbrich et al., 2022). Plants encountering biotic challenges in the form of either pathogen attack or herbivory synthesize and release phytohormones and VOCs, which amend the chemical composition of the root exudates and eventually restructure the below-ground microbial community (Erb et al., 2009; Dastogeer et al., 2020). Plants encountering various environmental stimuli either in physical or chemical form release a number of phytochemicals as root exudates, in contrast to the non-stimulated plants (Badri and Vivanco, 2009). Further, variable compounds are released by different plant species encountering the same stimulus at the same time. Several pieces of evidence suggest that the external application of defence-related compounds like SA, methyl jasmonate (MeJA), and nitric oxide (NO) triggers the host plant to accumulate a diverse range of secondary metabolites (Zhao et al., 2005; Badri and Vivanco, 2009), e.g., indole glucosinolates, rosmarinic acid, and caffeic acid (**Figure 1A**).

In response to recognition of pathogens and pests, plants induce SAR and ISR signalling that helps in defence priming and finally induces the host plant to respond robustly against upcoming pathogen attacks; e.g., SAR and ISR establish a memory mark by activating various defence-related genes or some protein factors like MAPK3 and MAPK6 (Beckers et al., 2009; Doornbos et al., 2012). As a result of biotic stress, root exudates become enriched with phenolic compounds, which can directly suppress the growth of pathogenic microbes (Jousset et al., 2011; Badri et al., 2013; Chaparro et al., 2013) or may indirectly act by changing the expression level of some anti-fungal genes present in the plant beneficial microorganisms (Jousset et al., 2011). For example, barley plants growing in split root systems infected with the pathogenic fungus *Pythium ultimum* in one side of the root and inoculation of the bacterium *Pseudomonas fluorescens* on the other side secrete root exudates having a more phenolic compound. This specialized exudation recruits beneficial PGPR towards the roots and inhibits the spore germination of *P. ultimum* and also results in the activation of antibiotics-related genes present in *P. fluorescens* against *P. ultimum* (Jousset et al., 2011). Thus, plants under biotic stress may induce either direct or indirect defence mechanisms, where the exudation of specialised compounds through the roots might have a positive effect.

*Capsicum annuum*, undergoing herbivory attack due to aphids and also infected with *Ralstonia solanacearum* SL1931, has shown the attraction of a higher population of beneficial bacteria *B. subtilis* GB03 in the rhizospheric region of the plant (Lee et al., 2012). Earlier experimental pieces of evidence suggested that the recruitment of *B. subtilis* FB17 towards the plant rhizospheric region might be due to the secretion of l-malic acid as root exudates (Rudrappa et al., 2008; Badri et al., 2009). *Pseudomonas syringae*-infected *A. thaliana* has resulted in the alteration of malic acid concentration by altering the gene expression of root malate transporter, which led to the accumulation of a large amount of the rhizobacterium *B. subtilis* at the rhizosphere (Lakshmanan et al., 2012; Chen et al., 2013; Dastogeer et al., 2020). Infection of cucumber plants with *Fusarium oxysporum* f. sp. *cucumerinum* amended the secretion of fumaric acid and citric acid at the rhizospheric region of the cucumber plants and helped in *Bacillus amyloliquefaciens* biofilm formation (Dastogeer et al., 2020). In the case of carex plant, *Fusarium culmorum* infection has resulted in the secretion of monoterpene (*Z*)-limonene-oxide in the root exudates and attracted a group of microbes including *Janthinobacterium*, *Collimonas*, and *Paenibacillus* towards the rhizospheric region (Schulz-Bohm et al., 2018). This indicates that a distinct blend of signalling molecules are involved in the recruitment of specific rhizobacterial communities. In the **Table 1**, salient findings from the studies conducted on biotic stress induced change in root exudate composition and modulation of microbial community are enumerated.

**Table 1:** Biotic factors modulating rhizospheric microbial community due to altered root exudate compositions.

Plant species	Biotic factors	Prime root exudate composition (signalling molecule)	Abundant rhizospheric soil microbial community	References
*Arabidopsis thaliana*	*Pseudomonas syringae* pv tomato (Pst)	Altered malic acid concentration	*Bacillus subtilis*	Lakshmanan et al., 2012; Chen et al., 2013; Dastogeer et al., 2020
		Amino acids and long-chain fatty acids	*Pseudomonas* sp.	Wen et al., 2021
Barley (*Hordeum vulgare*)	*Pythium ultimum*	Phenolic compound	*Pseudomonas fluorescens*	Jousset et al., 2011
Cucumber (*Cucumis sativus*)	*Fusarium oxysporum* f. sp. *cucumerinum*	Altered fumaric and citric acid concentration	*Bacillus amyloliquefaciens*	Dastogeer et al., 2020
Chilli (*Capsicum annuum*)	Aphids and *Ralstonia solanacearum* SL1931	l-Malic acid	*B. subtilis* GB03	Badri et al., 2009; Lee et al., 2012
Carex (*Carex arenaria*)	*Fusarium culmorum*	Monoterpene (*Z*)-limonene-oxide (volatile organic compound)	*Janthinobacterium*, *Collimonas*, and *Paenibacillus*	Schulz-Bohm et al., 2018
Oilseed rape (*Brassica napus*)	Cabbage root fly (*Delia radicum*)	Significant increase in trehalose, indolyl glucosinolates, and sulfur, including a decrease in the level of amino acids and sugar content	Gammaproteobacteria and Firmicutes also recruit *Bacillus*, *Paenibacillus*, *Pseudomonas*, and *Stenotrophomonas*	Ourry et al., 2018
Maize (*Zea mays* L.)	*Diabrotica virgifera* larvae	Sesquiterpene, (*E*)-*b*-caryophyllene (Ebc)	Soil-borne entomopathogenic nematode	Rasmann et al., 2005; Degenhardt et al., 2009
	Larvae of western corn rootworms	Phenolic compounds	*Acinetobacter calcoaceticus*	Dematheis et al., 2012
Peanut (*Arachis hypogaea* L.)	Cassava (*Manihot esculenta*)	Ethylene (volatile organic compounds)	Actinobaterial species (*Catenulispora*)	Chen et al., 2020
Sorghum (*Sorghum bicolor*)	*Striga hermonthica*	Strigolactone	AMF colonization	Lendzemo et al., 2007
Tomato (*Solanum lycopersicum*)	*Meloidogyne incognita*	Strigolactone	AMF colonization	Xu et al., 2019
	*Fusarium oxysporum*	Benzonitrile, benzothiazole, dimethyl trisulfide, formic acid, and a terpene-like compound (volatile organic compounds)	*Bacillus* sp.	Gulati et al., 2020

AMF, arbuscular mycorrhizal fungi.

Plants experience concurrent infestation by various biotrophic or necrotrophic insects in their natural habitat, ultimately inducing the SA or JA pathways for defence. Several studies have revealed that the SA and JA crosstalk may act synergistically or antagonistically (Mur et al., 2006; Wasternack and Hause, 2013). Further, when an herbivore or an insect attacks a plant, they release specific salivary effectors, which directly or indirectly influence the activation of SA or JA pathways (Xu et al., 2018). The molecular mechanisms involved in the regulation of SA and JA crosstalk are not yet very clear. However, according to the studies, NONEXPRESSER OF PATHOGENESIS-RELATED GENES1 (NPR1) acts as a key regulator in SA-induced defence gene expression during the attack of a biotrophic insect, while the SCF^COI1^ complex helps in the degradation of jasmonate-ZIM domain (JAZ), allowing the activation of JA-mediated defence-related genes (Xu et al., 2022). For example, potato plants infested with herbivores having varied feeding behaviour induce JA signalling during the infestation by necrotrophic insects, while SA signalling is induced in plants during the attack of biotrophic insects (Bari and Jones, 2009; Malacrinò et al., 2021). A deeper understanding of herbivore infestation with different feeding behaviours revealed that herbivore feeding strategies have a negligible effect on the rhizomicrobiota (Malacrinò et al., 2021). However, altogether herbivore-induced stress affects the root exudation pattern and results in the recruitment of beneficial microbes to alleviate biotic stress (Rasmann and Turlings, 2016; Hoysted et al., 2018; Rolfe et al., 2019). SA in the root exudates helps in the recruitment of siderophore-producing rhizobacteria and takes part in suppressing the disease development in the host plant; further, the threshold concentration of SA in the soil is sufficient to alert the neighbouring plants to withstand upcoming stress (Bakker et al., 2014; Aznar and Dellagi, 2015; Cheol Song et al., 2016; Rolfe et al., 2019). Yuan et al. (2018) inoculated *A. thaliana* plants with *P. syringae* pv tomato (Pst) and observed an increase in JA level and improvement in plant immune responses. Plants encountering Pst infection exhibited a significant increase in the amount of amino acids, nucleotides, and long-chain organic acids (LCOAs) and simultaneously decreased the amount of sugars, alcohols, and short-chain organic acids (SCOAs) in the root exudates (Li et al., 2021a; Wen et al., 2021). The modification in the root exudate composition changed the rhizosphere microbiome structure and elicited disease resistance characteristics in *A. thaliana*. This might be due to the recruitment of beneficial rhizobacterium *B. subtilis* under the influence of an altered root exudation pattern (Lakshmanan et al., 2012; Chen et al., 2013; Dastogeer et al., 2020). Strigolactone (SL) released as root exudates have a direct impact on structuring the root-associated microbiome, as it enhances AMF colonization in plant roots (Akiyama and Hayashi, 2006). SL synthesis was found to increase in the roots of tomato plants undergoing infestation of parasitic nematode *Meloidogyne incognita*, augmenting the root–AMF interactions for better resilience of tomato plants (Xu et al., 2019). Further, the use of SL analogue racGR24 in JA-deficient mutants of tomato enhanced plant defence against nematode infection. In addition to that, the secretion of specific molecules like benzonitrile, benzothiazole, dimethyl trisulfide, formic acid, and terpene-like compounds from the roots of tomato plants infected with *Fusarium oxysporum* was found to recruit *Bacillus* sp. in the rhizosphere. The recruitment of *Bacillus* sp. might be due to the change in root exudate composition (Gulati et al., 2020).

*Arabidopsis* plants treated with MAMP enhance the activation of defence genes CYP71A12 and MYB51 in the root region (Millet et al., 2010; Rolfe et al., 2019), having regulatory effects on the synthesis of tryptophan-derived compounds like camalexin and indolic glucosinolates. These molecules are commonly detected in root exudates and have a direct effect on regulating the rhizosphere microbiome, as they possess both antimicrobial and signalling activities (Stotz et al., 2011; Mönchgesang et al., 2016; Koprivova et al., 2019; Rolfe et al., 2019). Infestation of the *Diabrotica virgifera virgifera* larvae at the roots of maize plants trigger the secretion of the sesquiterpene, (*E*)-*b*-caryophyllene (Ebc), which results in the recruitment of soil-borne entomopathogenic nematode (Rasmann et al., 2005; Degenhardt et al., 2009). The entomopathogenic nematode attracted *via* the Ebc signalling acts as a natural predator for the beetle *D. v. virgifera*, showing biocontrol activity. However, Ebc secretion may not always act as a biocontrol strategy, as in a non-yielding maize line, Ebc accumulation in the root–soil junction induced hyphal branching of *Colletotrichum graminicola* and *Fusarium graminearum*, which are considered as the key fungal pathogen. Thus, Ebc secretion as root exudates might have the ability to either enhance plant resistance or induce plant susceptibility, depending upon the type of biotic disturbances (Fantaye et al., 2015). In various studies, it has been suggested that the overexpression of the Ebc gene might have a wide-ranging impact on soil microbes (Rolfe et al., 2019). In maize or other cereals, a class of secondary metabolites known as benzoxazinoids has a direct link with the induction of JA-dependent defence signalling against herbivore attack. Again, 6-methoxy-benzoxazolin-2-one (MBOA) and methoxy-2*H*-1,4-benzoxazin-3(4*H*)-one (DIMBOA), are the product obtained from the fractionation of benzoxazinoids and helps in the alteration of rhizosphere microbiome structure when released as a root exudate and even enhances the resistance of the next-generation plants against herbivory (Niemeyer, 2009; Hu et al., 2018). Further research is needed on both MBOA and DIMBOA exudation patterns concerning biotic stress and the role of both the compounds and their effects on soil and neighbouring plants. Dematheis et al. (2012) reported the abundance of *Acinetobacter calcoaceticus* in the rhizosphere of maize under the influence of the larvae of western corn rootworms, which elicits phenolic compounds as exudates and affects mainly the bacterial communities rather than the fungi. The threatening soil herbivorous insect cabbage root fly (*Delia radicum*) and its major susceptible host oilseed rape (*Brassica napus*) were used in a study to determine the effect of herbivory in shaping the root–rhizosphere microbiome. Infestation by the insect, attracted more numbers of Gammaproteobacteria and Firmicutes towards the root and rhizospheric region of oilseed rape and also enhanced the abundance of four bacterial groups, namely, *Bacillus*, *Paenibacillus*, *Pseudomonas*, and *Stenotrophomonas*. Modulation of the bacterial communities in the root and the rhizosphere was due to the increasing trehalose, indolyl glucosinolates, and sulfur including a decrease in the level of amino acids and sugar content in the root exudates (Ourry et al., 2018). Thus, under the influence of various biotic factors, plants release a diverse range of signalling molecules, ultimately shaping the rhizomicrobiota. Biotic factors influencing the rhizospheric microbial community may be due to changes in primary and secondary metabolite concentration gradients along the root and rhizosphere. The altered rhizospheric microbial community can in turn affect the plant root exudation pattern.

### Role of neighbours in the alteration of plant root exudation profiles

As described earlier, the rhizosphere serves as a dwelling place for an enormous number organisms interacting with roots, and it also shows the interaction of root systems with neighbouring plants (Badri et al., 2009). The exudates secreted from the roots establish a chemical crosstalk with the below-ground organisms as well as with their surrounding roots. Root exudates act in a multipurpose way, and this can be easily understood with the example of isoflavone exudation from the roots of soybean plants, as isoflavones secreted out from the soybean roots recruit both mutualistic (*Bradyrhizobium japonicum*) and pathogenic (*Phytophthora sojae*) microbes towards them (Bais et al., 2006). The actual mechanisms of how roots interpret a multitude of signals received from surroundings are not yet understood; however, the mechanism of root secretion is assumed (**Figure 1B**). Small polar and uncharged molecules of low molecular weight are transported through lipid membranes depending on the membrane permeability and pH of the cytosol. In contrast, the protein and ion channels help in the transportation of sugar molecules, amino acids, and carboxylate anions outside the cell membrane depending upon the electrochemical gradient. Compounds having high molecular weight are generally excreted by plants using vesicular transport into the rhizosphere (Badri and Vivanco, 2009). Plants have developed certain mechanisms to alleviate the toxic effect of heavy metals (namely, iron, zinc, manganese, and copper) present in the soil through metal homeostasis (Haydon and Cobbett, 2007). Nutrient accessibilities in the soil modulate the root exudation pattern, which results in changes in the soil pH and the formation of metabolite complexes with the metal ions (Chen et al., 2017). Plants secrete a blend of complex metabolites comprising gaseous molecules, inorganic ions, and carbon-based compounds for the regulation of nutrient availability and to detoxify intolerable metal pollutants in their surroundings. The type of secretion varies among species depending upon species tolerance to nutrient deficiency and heavy metal contamination. Plants use two strategies for the uptake of required nutrients using complex metabolite compositions: reduction-based strategy and chelation-based strategy (Chen et al., 2017). For example, Fe deficiency induces secretion of riboflavin (Rbfl), phenolics, and phytosiderophore compounds *via* the upregulation of certain enzymes (dimethyl-8-ribityllumazine synthase (DMRL) and GTP cyclohydrolase II (GTPcII) required for flavin biosynthesis) (Rellán-Álvarez et al., 2010, Rodriguez-Clema et al., 2011, Rodríguez-Celma et al., 2013; Hsieh and Waters, 2016), transcription factors (bHLH38 in *Arabidopsis* and bHLH39 in tobacco and sunflower reported for the Rbfl accumulation and exudation) (Vorwieger et al., 2007), and plasma membrane-localized transporters (pleiotropic drug resistance 9 (PDR9) for phenolics and transporter of mugineic acid1 (TOM1) for phytosiderophores) (Nozoye T et al., 2011, Nozoye T et al., 2013; Fourcroy et al., 2014; Chen et al., 2017).

Recent evidence showed that the secretion of proteins as root exudates is regulated by the microorganisms present in the soil. The presence of a specific microbial community in the rhizosphere can alter the protein constituents of root exudates and vice versa (De-la-Peña et al., 2008). An experiment was conducted with two plants, namely, *A. thaliana* and *Medicago sativa*, by using *P. syringae* (DC3000) and *Sinorhizobium meliloti* (RM1021) as a pathogen and a symbiont, respectively. Secretions of seven different proteins (categorized as hydrolases, peptidases, and peroxidases) were reported during *Medicago*–*S. meliloti* interactions, but no such secretion had been observed when the *Medicago* plants were exposed to *P. syringae*. Similarly, *Arabidopsis*–*P. syringae* interaction had led to the exudation of several defence-related proteins, but like the alternative result of *Medicago*–*P. syringae*, no defence molecules have been secreted during the interaction of *Arabidopsis* and *S. meliloti*. This host–microbe-specific result was observed due to the differences in root exudate composition, mainly the protein components secreted out by both the host plants under variable plant–microbe interaction (De-la-Peña et al., 2008; Badri and Vivanco, 2009). Additionally, several studies have also reported the effect of weeds on agricultural fields, where they inhibit the proper growth and development of domesticated plant species. When the weeds migrate from their native region to a new place, they become more invasive and have a consequent effect on the domesticated plant species. As the weeds become exotic, they escape their specialized insects and pathogens including other factors at their native place and change their physiology and biochemistry to become more resilient. Weeds can alter the root exudation profile, which exhibits allelopathy to native plants as well as ensures the recruitment of beneficial microbes (Trognitz et al., 2016; Trivedi et al., 2022). Various rhizospheric microbes contribute to lowering the effect of allelopathy (Mishra et al., 2012a; Mishra et al., 2012b; Trognitz et al., 2016); e.g., invasive plants like *Parthenium hysterophorus* are widely found in India and some other countries, which secrete parthenin and phenolic acids as allelopathic chemicals. *Pseudomonas putida* NBRIC19 strain modulates the composition of antioxidant enzymes in root exudates of *P. hysterophorus* and alleviates the allelopathic effect (Mishra and Nautiyal, 2012; Mishra et al., 2012a; Mishra et al., 2012b). In a population having a large number of *Arabidopsis* plants, enhanced secretion of glucosinolates was observed, as a result of the neighbouring plant’s effect. However, the secretion of glucosinolates was reduced in an environment with a smaller number of *Arabidopsis* plants (Wentzell and Kliebenstein, 2008). Similarly, Bressan et al. (2009) in their study, using denaturing gradient gel electrophoresis (DGGE) fingerprint analysis, unearthed the impact of exogenously produced glucosinolate by *A. thaliana* transgenic line CYP79A1 on rhizospheric soil microbial community where an abundance of *Agrobacterium* sp., *Bosea* sp., *Rhizobium* sp., *Mesorhizobium amorphae*, and *Syncephalis depressa* in the rhizospheric region has been found as compared to the wild-type plants. Further, intercropping of peanut (*Arachis hypogaea* L.) with cassava (*Manihot esculenta* Crantz) plants triggers the production of ethylene hormone by the peanut plants. The release of ethylene acts as a signalling molecule in the rhizosphere and recruits the actinobacterial species *Catenulispora* towards the peanut roots, enhancing plant fitness in the intercropped area (Chen et al., 2020). These pieces of evidence suggest that neighbouring plants modulate each other’s exudation patterns and reshape the rhizosphere microbiome, leading to enhanced adaptation of plants under varied environmental conditions.

### The contemporary art of below-ground exudations

The rhizosphere harbours infinite numbers of microorganisms and other chemical substances along with plant roots. Complex ecological processes in the rhizosphere involve positive and negative interactions between the microfauna, plant–plant, or plant–microbes, which play a key role in maintaining the soil characteristics (Bais et al., 2006). The exudate secreted out by the roots influence both positive and negative interactions in the rhizosphere (Li et al., 2021b). One of the major constituents of root exudates is the VOCs, which play a critical role in shaping the below-ground microbiota. The VOCs can aid in the detection of neighbours, maintain a homeostatic interaction among neighbouring plants, and enhance plants’ adaptive capabilities under various circumstances (Ninkovic et al., 2016). Further, the rhizobacterial-released VOCs (carbon-based compounds, terpenoids, and sulfur compounds) are well-known organic compounds playing a crucial role in inter and intra specific communications below-ground (Mhlongo et al., 2018). Root exudates can promote plant growth by synergistically acting with the microbes, plants, and soil-dwelling nematodes forming a “tritrophic interaction” (Badri and Vivanco, 2009). Horiuchi et al. (2005), in their study, reported that root exudates of leguminous plants attract the nematode *Caenorhabditis elegans*, which is a vector as well as the symbiont of *S. meliloti*. The accumulation of *C. elegans* along with *S. meliloti* triggers the host plant to release VOCs, and eventually, the volatiles released recruit rhizobial bacteria, which in turn increases nodule formation in the legumes. Furthermore, insect-infested maize roots released specialized root exudates having Ebc as a major constituent and recruited entomopathogenic nematodes in the rhizosphere (Rasmann et al., 2005). Another example of tritrophic interaction including the host plant (Sorghum), AMF, and parasitic plant *Striga hermonthica* shows how the root exudate-mediated AMF colonization leads to the inhibition of parasitic plants (Lendzemo et al., 2007). The exudation of strigolactones and their derivatives play an important role in regulating this tritrophic interaction (Bouwmeester et al., 2007; Lendzemo et al., 2007). Therefore, it can be hypothesised that a tritrophic interaction leads to the knockout of harmful microbes by attracting beneficial microorganisms with the help of root exudates.

The rapid evolution of the rhizospheric microbes along a parasitic–mutualistic continuum is evident in some cases, in which root exudates are considered as a determining factor (Li et al., 2021a). Domesticated plants in their native areas interact with diverse co-evolved microorganisms, which may be either beneficial or harmful in nature. However, it has been observed that domesticated plants flourish effectively in an introduced area due to a smaller number of pathogens present in the soil, but if some foreign pathogens enter the ecosystem, it becomes dominant and highly destructive against the entire crop species, as there are no co-evolved competitors against them. The famous potato famine of Ireland (1845-52) is a well-known example of this situation, where the fungus *Phytophthora infestans* caused havoc in the potato fields of Ireland (Badri and Vivanco, 2009). Exploring the root exudate composition of wild and invasive plants might help in deciphering novel root exudate traits for sustainable agriculture.

## Root exudate composition according to plant’s mode of photosynthesis

**Figure 2:**
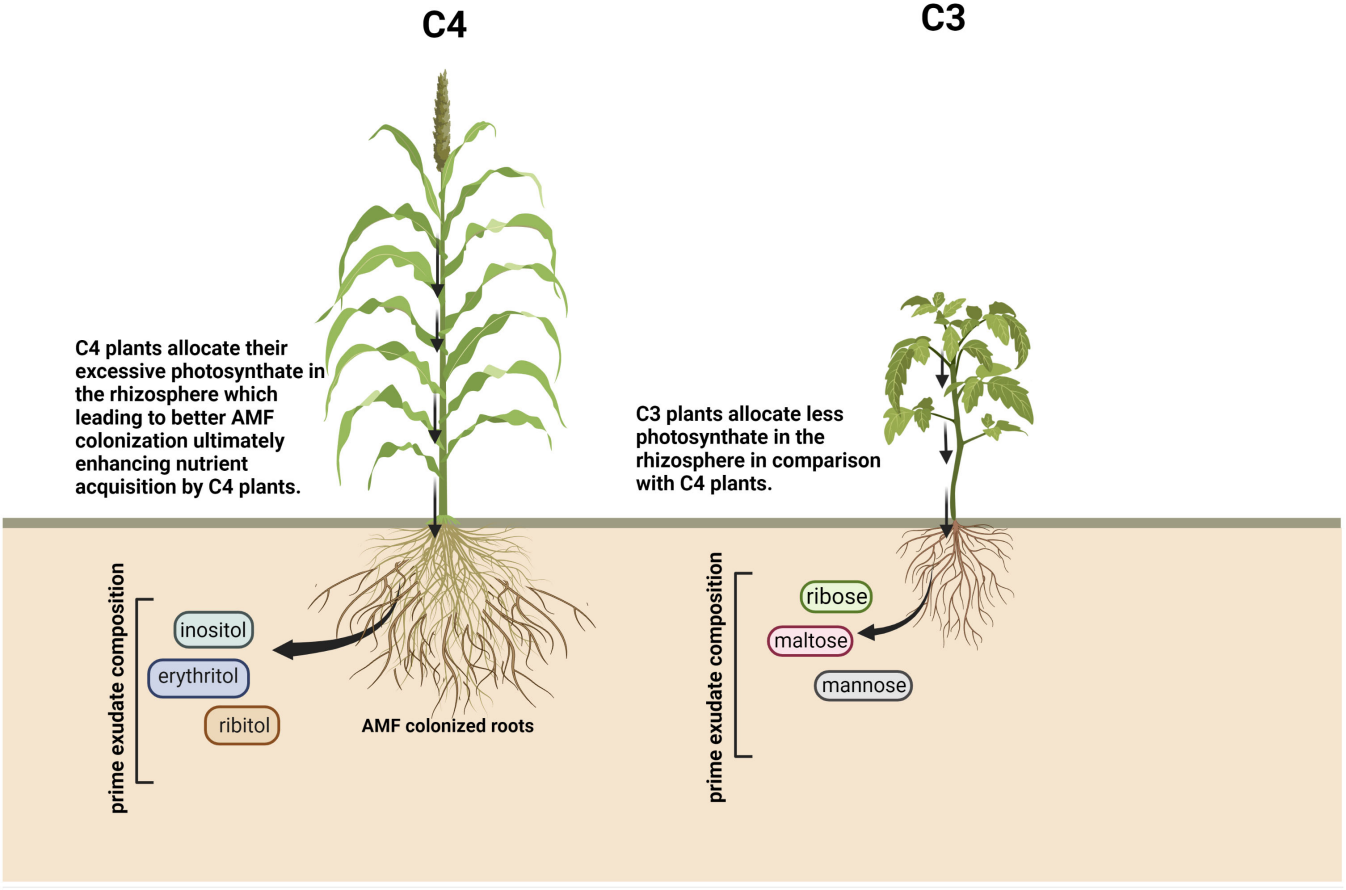
Schematic representation of root exudate compositions according to plants’ mode of photosynthesis. Created with BioRender.com.

Photosynthetic machinery involving C3, C4, and CAM pathways categorizes the plants based on the specific pathway used during photosynthesis. The functioning mechanism of C3 and C4 plants vary based on the site of carbon fixation, and the initial stable compound forms and acts as a substrate during the dark reaction of photosynthesis, where C3 plants convert three carbon molecules (phosphoglycerate) and C4 plants convert four carbon molecules (oxaloacetate) into metabolically active organic compounds. The correlation between the root exudation pattern and the plant’s mode of photosynthesis shows that root exudate composition differs depending upon the mode of photosynthesis. The major constituents of root exudates vary in both the plant groups, and it has been found that C3 plants release a large amount of carbohydrates and organic carbons (Nabais et al., 2011), whereas C4 plants mostly secrete amino acids and organic acids as a major constituent of root exudates. In C3 plants, mainly mannose, maltose, and ribose constitute the exudates (Vranova et al., 2013), but in C4 plants, inositol, erythritol, and ribitol are found (Olanrewaju et al., 2019) (**Figure 2**). Variability in the exudation pattern of C3 and C4 plants might have contrasting effects in shaping the rhizospheric microbial community. C4 plants in comparison to C3 plants are more tolerant to adverse environmental situations, but the role played by root exudates in C4 plants’ adaptability is not yet clear. It is evident that under elevated CO_2_ levels, C4 plants allocate their excessive photosynthates in the rhizosphere with the help of root exudation, which leads to better AMF colonization finally resulting in enhanced nutrient acquisition by C4 plants (Frew and Price, 2019), which indicates the reason behind the better resilience of C4 plants in stress condition. Additionally, a large proportion of weeds fall under the category of C4 plants, exhibiting a wide range of adaptive behaviour under various environmental perturbations. Weeds secrete allelochemicals as their root exudates, attracting more beneficial microbes and resulting in better adaptation than the neighbouring crop plants (Trognitz et al., 2016; Trivedi et al., 2022).

C4 plants, like maize, induce JA-dependent resistance against herbivores (*Spodoptera littoralis*) due to the exudation of BX from the roots, which changes the rhizospheric microbial structure and makes the maize plants more resistant against the caterpillar attack (Hu et al., 2018). In contrast, tobacco plants infected with black shank disease caused by *Phytophthora nicotianae* can perform a pre-infection prevention strategy by exuding antimicrobial substances and lead to increased disease resistance by eliciting plant defence responses (Zhang et al., 2020). There are very few reports on studies elucidating the composition of the exudates according to the plant’s mode of photosynthesis and their influence on the rhizospheric microbial community. To have a comprehensive understanding, future research work on root exudate secretion mechanisms in C3 and C4 plants is warranted.

## Conclusion and future perspectives

The secretion of root exudates as a signalling molecule can help in deciphering the plant’s stress status with the rhizospheric microbes and the neighbouring plants. Root exudates serve as organic carbon sources for rhizospheric microbes and their composition changes during plants’ interaction with pathogens or neighbouring plants either at above-ground or below-ground levels. Specific exudates are released under biotic stress conditions, thereby altering rhizospheric microbial community structure and promoting plant health by inducing SAR or ISR. Maintenance of a low-entropy state in the rhizosphere is important for creating equilibrium and promoting root–soil–microbe interactions by maximizing energy utilization, which optimizes localized nutrient supply. Further, a detailed understanding of entropy-based modulation of root exudates and the rhizomicrobiota in crops and crop wild relatives (CWRs) might pave the way for sustainable crop management. With the advances in molecular biology techniques, the root exudate compositions of various plants might be studied in detail under natural conditions. In addition, traits associated with root exudation of CWRs might have effective agronomic use in the future, as such type of traits might be exploited in the future to generate pest-resistant, fertilizer-independent, or weed-suppressive crops (Preece and Peñuelas, 2020). Furthermore, exploration of the rhizosphere microbiome structure of C3 and C4 plants under varied biotic stresses will help in designing specific agronomic strategies for these groups of plants. The molecular mechanisms governing the root exudation process are not very clear to date, but a detailed understanding of root exudation can provide a solution for effective stress management.

## Author contributions

NA was responsible for writing guidance and manuscript preparation. IS and SK were responsible for writing the first draft of the review article. NA revised the article with IS and SK. All authors contributed to the article and approved the submitted version.

## References

[B1] Afridi M. S. Fakhar A. Kumar A. Ali S. Medeiros F. H. Muneer M. A.. (2022). Harnessing microbial multitrophic interactions for rhizosphere microbiome engineering. Microbiol. Res. 265, 127199. doi: 10.1016/j.micres.2022.127199 36137486

[B2] Akiyama K. Hayashi H. (2006). Strigolactones: Chemical signals for fungal symbionts and parasitic weeds in plant roots. Ann. Bot. 97 (6), 925–931. doi: 10.1093/aob/mcl063 16574693 PMC2803390

[B3] Aznar A. Dellagi A. (2015). New insights into the role of siderophores as triggers of plant immunity: what can we learn from animals? J. Exp. Bot. 66 (11), 3001–3010. doi: 10.1093/jxb/erv155 25934986

[B4] Badri D. V. Chaparro J. M. Zhang R. Shen Q. Vivanco J. M. (2013). Application of natural blends of phytochemicals derived from the root exudates of arabidopsis to the soil reveal that phenolic-related compounds predominantly modulate the soil microbiome. J. Biol. Chem. 288 (7), 4502–4512. doi: 10.1074/jbc.M112.433300 23293028 PMC3576057

[B5] Badri D. V. Vivanco J. M. (2009). Regulation and function of root exudates. Plant Cell Environ. 32 (6), 666–681. doi: 10.1111/j.1365-3040.2009.01926.x 19143988

[B6] Badri D. V. Weir T. L. van der Lelie D. Vivanco J. M. (2009). Rhizosphere chemical dialogues: Plant–microbe interactions. Curr. Opin. Biotechnol. 20 (6), 642–650. doi: 10.1016/j.copbio.2009.09.014 19875278

[B7] Bais H. P. Weir T. L. Perry L. G. Gilroy S. Vivanco J. M. (2006). The role of root exudates in rhizosphere interactions with plants and other organisms. Annu. Rev. Plant Biol. 57 (1), 233–266. doi: 10.1146/annurev.arplant.57.032905.105159 16669762

[B8] Bakker P. A. Berendsen R. L. Van Pelt J. A. Vismans G. Yu K. Li E.. (2020). The soil-borne identity and microbiome-assisted agriculture: Llooking back to the future. Mol. Plant 13 (10), 1394–1401. doi: 10.1016/j.molp.2020.09.017 32979564

[B9] Bakker P. A. Ran L. Mercado-Blanco J. (2014). Rhizobacterial salicylate production provokes headaches! Plant Soil 382 (1), 1–16. doi: 10.1007/s11104-014-2102-0

[B10] Baluška F. Mancuso S. (2013). Root apex transition zone as oscillatory zone. Front. Plant Sci. 4. doi: 10.3389/fpls.2013.00354 PMC3788588 24106493

[B11] Bari R. Jones J. D. (2009). Role of plant hormones in plant defence responses. Plant Mol. Biol. 69 (4), 473–488. doi: 10.1007/s11103-008-9435-0 19083153

[B12] Battey N. H. Blackbourn H. D. (1993). The control of exocytosis in plant cells. New Phytol. 125 (2), 307–338. doi: 10.1111/j.1469-8137.1993.tb03883.x 33874503

[B13] Beckers G. J. Jaskiewicz M. Liu Y. Underwood W. R. He S. Y. Zhang S.. (2009). Mitogen-activated protein kinases 3 and 6 are required for full priming of stress responses in arabidopsis thaliana. Plant Cell 21 (3), 944–953. doi: 10.1105/tpc.108.062158 19318610 PMC2671697

[B14] Bednarek P. Osbourn A. (2009). Plant-microbe interactions: chemical diversity in plant defense. Science 324 (5928), 746–748.doi: 10.1126/science.1171661 19423814

[B15] Berendsen R. L. Pieterse C. M. Bakker P. A. (2012). The rhizosphere microbiome and plant health. Trends Plant Sci. 17 (8), 478–486. doi: 10.1016/j.tplants.2012.04.001 22564542

[B16] Bhutia D. D. Belbase S. Paudel J. Kumar S. (2023). “Plant exudates and microbial interaction–a change in dynamics,” in Climate change and microbiome dynamics: Carbon cycle feedbacks (Cham: Springer International Publishing), 83–95.

[B17] Bouwmeester H. J. Roux C. Lopez-Raez J. A. Becard G. (2007). Rhizosphere communication of plants, parasitic plants and AM fungi. Trends Plant Sci. 12 (5), 224–230. doi: 10.1016/j.tplants.2007.03.009 17416544

[B18] Bressan M. Roncato M. A. Bellvert F. Comte G. Haichar F. E. Z. Achouak W.. (2009). Exogenous glucosinolate produced by arabidopsis thaliana has an impact on microbes in the rhizosphere and plant roots. ISME J. 3 (11), 1243–1257. doi: 10.1038/ismej.2009.68 19554039

[B19] Brian G. Lea P. J. (2007). Glutamate in plants: Metabolism, regulation, and signaling. J. Exp. Bot. 58 (9), 2339–2358. doi: 10.1093/jxb/erm121 17578865

[B20] Bulgarelli D. Rott M. Schlaeppi K. Ver Loren van Themaat E. Ahmadinejad N. Assenza F.. (2012). Revealing structure and assembly cues for arabidopsis root-inhabiting bacterial microbiota. Nature 488 (7409), 91–95. doi: 10.1038/nature11336 22859207

[B21] Canarini A. Kaiser C. Merchant A. Richter A. Wanek W. (2019). Root exudation of primary metabolites: Mechanisms and their roles in plant responses to environmental stimuli. Front. Plant Sci. 10, 157. doi: 10.3389/fpls.2019.00157 30881364 PMC6407669

[B22] Chaparro J. M. Badri D. V. Bakker M. G. Sugiyama A. Manter D. K. Vivanco J. M. (2013). Root exudation of phytochemicals in arabidopsis follows specific patterns that are developmentally programmed and correlate with soil microbial functions. PloS One 8 (2), e55731. doi: 10.1371/annotation/51142aed-2d94-4195-8a8a-9cb24b3c733b 23383346 PMC3562227

[B23] Chater K. F. Biró S. Lee K. J. Palmer T. Schrempf H. (2010). The complex extracellular biology of streptomyces. FEMS Microbiol. Rev. 34 (2), 171–198. doi: 10.1111/j.1574-6976.2009.00206.x 20088961

[B24] Chen Y. T. Wang Y. Yeh K. C. (2017). Role of root exudates in metal acquisition and tolerance. Curr. Opin. Plant Biol. 39, 66–72. doi: 10.1016/j.pbi.2017.06.004 28654805

[B25] Chen Y. Yan F. Chai Y. Liu H. Kolter R. Losick R.. (2013). Biocontrol of tomato wilt disease by b acillus subtilis isolates from natural environments depends on conserved genes mediating biofilm formation. Environ. Microbiol. 15 (3), 848–864. doi: 10.1111/j.1462-2920.2012.02860.x 22934631 PMC3904073

[B26] Chen Y. Bonkowski M. Shen Y. Griffiths B. S. Jiang Y. Wang X.. (2020). Root ethylene mediates rhizosphere microbial community reconstruction when chemically detecting cyanide produced by neighbouring plants. Microbiome 8 (1), 1–17. doi: 10.1186/s40168-019-0775-6 31954405 PMC6969408

[B27] Cheol Song G. Sim H. J. Kim S. G. Ryu C. M. (2016). Root-mediated signal transmission of systemic acquired resistance against above-ground and below-ground pathogens. Ann. Bot. 118 (4), 821–831. doi: 10.1093/aob/mcw152 27555496 PMC5055637

[B28] Dastogeer K. M. Tumpa F. H. Sultana A. Akter M. A. Chakraborty A. (2020). Plant microbiome–an account of the factors that shape community composition and diversity. Curr. Plant Biol. 23, 100161. doi: 10.1016/j.cpb.2020.100161

[B29] Degenhardt J. Hiltpold I. Köllner T. G. Frey M. Gierl A. Gershenzon J.. (2009). Restoring a maize root signal that attracts insect-killing nematodes to control a major pest. Proc. Natl. Acad. Sci. 106 (32), 13213–13218. doi: 10.1073/pnas.0906365106 19666594 PMC2726344

[B30] De-la-Peña C. Lei Z. Watson B. S. Sumner L. W. Vivanco J. M. (2008). Root-microbe communication through protein secretion. J. Biol. Chem. 283 (37), 25247–25255. doi: 10.1074/jbc.M801967200 18635546

[B31] de la Porte A. Schmidt R. Yergeau É. Constant P. (2020). A gaseous milieu: extending the boundaries of the rhizosphere. Trends Microbiol. 28 (7), 536–542. doi: 10.1016/j.tim.2020.02.016 32544440

[B32] Dematheis F. Zimmerling U. Flocco C. Kurtz B. Vidal S. Kropf S.. (2012). Multitrophic interaction in the rhizosphere of maize: Root feeding of western corn rootworm larvae alters the microbial community composition. PloS One 7 (5), e37288. doi: 10.1371/journal.pone.0037288 22629377 PMC3358342

[B33] De Schepper V. De Swaef T. Bauweraerts I. Steppe K. (2013). Phloem transport: A review of mechanisms and controls. J. Exp. Bot. 64 (16), 4839–4850. doi: 10.1093/jxb/ert302 24106290

[B34] Doornbos R. F. van Loon L. C. Bakker P. A. (2012). Impact of root exudates and plant defense signaling on bacterial communities in the rhizosphere. a review. Agron. Sustain. Dev. 32 (1), 227–243. doi: 10.1007/s13593-011-0028-y

[B35] Dowarah B. Gill S. S. Agarwala N. (2021). Arbuscular mycorrhizal fungi in conferring tolerance to biotic stresses in plants. J. Plant Growth Regul., 1–16. doi: 10.1007/s00344-021-10392-5

[B36] Du Y. Zou W. Zhang K. Ye G. Yang J. (2020). Advances and applications of clostridium co-culture systems in biotechnology. Front. Microbiol. 11, 560223. doi: 10.3389/fmicb.2020.560223 33312166 PMC7701477

[B38] Erb M. Lenk C. Degenhardt J. Turlings T. C. (2009). The underestimated role of roots in defense against leaf attackers. Trends Plant Sci. 14 (12), 653–659. doi: 10.1016/j.tplants.2009.08.006 19736036

[B39] Fantaye C. A. Köpke D. Gershenzon J. Degenhardt J. (2015). Restoring (E)-β-caryophyllene production in a non-producing maize line compromises its resistance against the fungus colletotrichum graminicola. J. Chem. Ecol. 41 (3), 213–223. doi: 10.1007/s10886-015-0556-z 25893788

[B40] Farrar J. F. Jones D. L. (2000). The control of carbon acquisition by roots. New Phytol. 147 (1), 43–53. doi: 10.1046/j.1469-8137.2000.00688.x

[B41] Fernández I. Cosme M. Stringlis I. A. Yu K. de Jonge R. van Wees S. M.. (2019). Molecular dialogue between arbuscular mycorrhizal fungi and the nonhost plant arabidopsis thaliana switches from initial detection to antagonism. New Phytol. 223 (2), 867–881. doi: 10.1111/nph.15798 30883790

[B42] Fierer N. Bradford M. A. Jackson R. B. (2007). Toward an ecological classification of soil bacteria. Ecology 88 (6), 1354–1364. doi: 10.1890/05-1839 17601128

[B43] Fourcroy P. Sisó-Terraza P. Sudre D. Savirón M. Reyt G. Gaymard F.. (2014). Involvement of the ABCG 37 transporter in secretion of scopoletin and derivatives by arabidopsis roots in response to iron deficiency. New Phytol. 201 (1), 155–167. doi: 10.1111/nph.12471 24015802

[B44] Frew A. Price J. N. (2019). Mycorrhizal-mediated plant–herbivore interactions in a high CO2 world. Funct. Ecol. 33 (8), 1376–1385. doi: 10.1111/1365-2435.13347

[B45] Garbeva P. Van Elsas J. D. Van Veen J. A. (2008). Rhizosphere microbial community and its response to plant species and soil history. Plant Soil 302 (1), 19–32. doi: 10.1007/s11104-007-9432-0

[B46] Gulati S. Ballhausen M. B. Kulkarni P. Grosch R. Garbeva P. (2020). A non-invasive soil-based setup to study tomato root volatiles released by healthy and infected roots. Sci. Rep. 10 (1), 1–11. doi: 10.1038/s41598-020-69468-z 32728091 PMC7391657

[B47] Haldar S. Sengupta S. (2015). Plant-microbe cross-talk in the rhizosphere: insight and biotechnological potential. Open Microbiol. J. 9, 1. doi: 10.2174/1874285801509010001 25926899 PMC4406998

[B48] Haydon M. J. Cobbett C. S. (2007). Transporters of ligands for essential metal ions in plants. New Phytol. 174 (3), 499–506. doi: 10.1111/j.1469-8137.2007.02051.x 17447906

[B49] Hofmeister A. E. Grabowski R. Linder D. Buckler W. (1993). L-serine and l-threonine dehydratase from clostridium propionicum two enzymes with different prosthetic groups. Eur. J. Biochem. 215 (2), 341–349. doi: 10.1111/j.1432-1033.1993.tb18040.x 8344301

[B50] Horiuchi J. I. Prithiviraj B. Bais H. P. Kimball B. A. Vivanco J. M. (2005). Soil nematodes mediate positive interactions between legume plants and rhizobium bacteria. Planta 222 (5), 848–857. doi: 10.1007/s00425-005-0025-y 16025342

[B51] Hoysted G. A. Bell C. A. Lilley C. J. Urwin P. E. (2018). Aphid colonization affects potato root exudate composition and the hatching of a soil borne pathogen. Front. Plant Sci. 9, 1278. doi: 10.3389/fpls.2018.01278 30237805 PMC6136236

[B52] Hsieh E. J. Waters B. M. (2016). Alkaline stress and iron deficiency regulate iron uptake and riboflavin synthesis gene expression differently in root and leaf tissue: implications for iron deficiency chlorosis. J. Exp. Bot. 67 (19), 5671–5685. doi: 10.1093/jxb/erw328 27605716 PMC5066488

[B53] Hu D. Baskin J. M. Baskin C. C. Liu R. Yang X. Huang Z. (2021). A seed mucilage-degrading fungus from the rhizosphere strengthens the plant-Soil-Microbe continuum and potentially regulates root nutrients of a cold desert shrub. Mol. Plant-Microbe Interact. 34 (5), 538–546. doi: 10.1094/MPMI-01-21-0014-FI 33596107

[B54] Hu L. Robert C. A. Cadot S. Zhang X. I. Ye M. Li B.. (2018). Root exudate metabolites drive plant-soil feedbacks on growth and defense by shaping the rhizosphere microbiota. Nat. Commun. 9 (1), 1–13. doi: 10.1038/s41467-018-05122-7 30013066 PMC6048113

[B55] İnceoğlu Ö. Falcão Salles J. van Elsas J. D. (2012). Soil and cultivar type shape the bacterial community in the potato rhizosphere. Microb. Ecol. 63 (2), 460–470. doi: 10.1007/s00248-011-9930-8 21898103 PMC3275741

[B56] Jousset A. Rochat L. Lanoue A. Bonkowski M. Keel C. Scheu S. (2011). Plants respond to pathogen infection by enhancing the antifungal gene expression of root-associated bacteria. Mol. Plant-Microbe Interact. 24 (3), 352–358. doi: 10.1094/MPMI-09-10-0208 21077773

[B57] Koprivova A. Schuck S. Jacoby R. P. Klinkhammer I. Welter B. Leson L.. (2019). Root-specific camalexin biosynthesis controls the plant growth-promoting effects of multiple bacterial strains. Proc. Natl. Acad. Sci. 116 (31), 15735–15744. doi: 10.1073/pnas.1818604116 31311863 PMC6681745

[B58] Lakshmanan V. Kitto S. L. Caplan J. L. Hsueh Y. H. Kearns D. B. Wu Y. S.. (2012). Microbe-associated molecular patterns-triggered root responses mediate beneficial rhizobacterial recruitment in arabidopsis. Plant Physiol. 160 (3), 1642–1661. doi: 10.1104/pp.112.200386 22972705 PMC3486800

[B59] Lauber C. L. Hamady M. Knight R. Fierer N. (2009). Pyrosequencing-based assessment of soil pH as a predictor of soil bacterial community structure at the continental scale. Appl. Environ. Microbiol. 75 (15), 5111–5120. doi: 10.1128/AEM.00335-09 19502440 PMC2725504

[B60] Lee B. Lee S. Ryu C. M. (2012). Foliar aphid feeding recruits rhizosphere bacteria and primes plant immunity against pathogenic and non-pathogenic bacteria in pepper. Ann. Bot. 110 (2), 281–290. doi: 10.1093/aob/mcs055 22437662 PMC3394643

[B61] Lendzemo V. W. Kuyper T. W. Matusova R. Bouwmeester H. J. Ast A. V. (2007). Colonization by arbuscular mycorrhizal fungi of sorghum leads to reduced germination and subsequent attachment and emergence of striga hermonthica. Plant Signaling Behav. 2 (1), 58–62. doi: 10.4161/psb.2.1.3884 PMC2633899 19516969

[B62] Li E. de Jonge R. Liu C. Jiang H. Friman V. P. Pieterse C. M.. (2021a). Rapid evolution of bacterial mutualism in the plant rhizosphere. Nat. Commun. 12 (1), 1–13. doi: 10.1038/s41467-021-24005-y 34158504 PMC8219802

[B63] Li J. Wang C. Liang W. Liu S. (2021b). Rhizosphere microbiome: The emerging barrier in plant-pathogen interactions. Front. Microbiol. 12, 772420. doi: 10.3389/fmicb.2021.772420 34777326 PMC8586421

[B64] Ling N. Wang T. Kuzyakov Y. (2022). Rhizosphere bacteriome structure and functions. Nat. Commun. 13 (1), 1–13. doi: 10.1038/s41467-022-28448-9 35149704 PMC8837802

[B65] Luo H. Xu H. Chu C. He F. Fang S. (2020). High temperature can change root system architecture and intensify root interactions of plant seedlings. Front. Plant Sci. 11, 160. doi: 10.3389/fpls.2020.00160 32161613 PMC7054236

[B66] Malacrinò A. Wang M. Caul S. Karley A. J. Bennett A. E. (2021). Herbivory shapes the rhizosphere bacterial microbiota in potato plants. Environ. Microbiol. Rep. 13 (6), 805–811. doi: 10.1111/1758-2229.12998 34427053

[B67] Massalha H. Korenblum E. Malitsky S. Shapiro O. H. Aharoni A. (2017). Live imaging of root–bacteria interactions in a microfluidics setup. Proc. Natl. Acad. Sci. 114 (17), 4549–4554. doi: 10.1073/pnas.1618584114 28348235 PMC5410799

[B68] Meyer S. De Angeli A. Fernie A. R. Martinoia E. (2010). Intra-and extra-cellular excretion of carboxylates. Trends Plant Sci. 15 (1), 40–47. doi: 10.1016/j.tplants.2009.10.002 19913451

[B69] Mhlongo M. I. Piater L. A. Madala N. E. Labuschagne N. Dubery I. A. (2018). The chemistry of plant–microbe interactions in the rhizosphere and the potential for metabolomics to reveal signaling related to defense priming and induced systemic resistance. Front. Plant Sci. 9, 112. doi: 10.3389/fpls.2018.00112 29479360 PMC5811519

[B70] Millet Y. A. Danna C. H. Clay N. K. Songnuan W. Simon M. D. Werck-Reichhart D.. (2010). Innate immune responses activated in arabidopsis roots by microbe-associated molecular patterns. Plant Cell 22 (3), 973–990. doi: 10.1105/tpc.109.069658 20348432 PMC2861455

[B71] Mishra S. Chauhan P. S. Goel A. K. Upadhyay R. S. Nautiyal C. S. (2012a). Pseudomonas putida NBRIC19 provides protection to neighboring plant diversity from invasive weed parthenium hysterophorus l. by altering soil microbial community. Acta physiol. plantarum 34 (6), 2187–2195. doi: 10.1007/s11738-012-1019-6

[B72] Mishra S. Mishra A. Chauhan P. S. Mishra S. K. Kumari M. Niranjan A.. (2012b). Pseudomonas putida NBRIC19 dihydrolipoamide succinyltransferase (SucB) gene controls degradation of toxic allelochemicals produced by parthenium hysterophorus. J. Appl. Microbiol. 112 (4), 793–808. doi: 10.1111/j.1365-2672.2012.05256.x 22324517

[B73] Mishra S. Nautiyal C. S. (2012). Reducing the allelopathic effect of parthenium hysterophorus l. @ on wheat (Triticum aestivum l.) by pseudomonas putida. Plant Growth Regul. 66 (2), 155–165. doi: 10.1007/s10725-011-9639-1

[B74] Mönchgesang S. Strehmel N. Schmidt S. Westphal L. Taruttis F. Müller. (2016). Natural variation of root exudates in arabidopsis thaliana-linking metabolomic and genomic data. Sci. Rep. 6 (1), 1–11. doi: 10.1038/srep29033 27363486 PMC4929559

[B75] Münch E. (1930). Die stoffbewegungen in der pflanze (Gustav Fischer, jena). Curr. Opin. Plant Biol. 43, 36–42.

[B76] Mur L. A. Kenton P. Atzorn R. Miersch O. Wasternack C. (2006). The outcomes of concentration-specific interactions between salicylate and jasmonate signaling include synergy, antagonism, and oxidative stress leading to cell death. Plant Physiol. 140 (1), 249–262. doi: 10.1104/pp.105.072348 16377744 PMC1326048

[B77] Nabais C. Labuto G. Gonçalves S. Buscardo E. Semensatto D. Nogueira A. R. A.. (2011). Effect of root age on the allocation of metals, amino acids and sugars in different cell fractions of the perennial grass paspalum notatum (bahiagrass). Plant Physiol. Biochem. 49 (12), 1442–1447. doi: 10.1016/j.plaphy.2011.09.010 22078382

[B78] Nazoa P. Vidmar J. J. Tranbarger T. J. Mouline K. Damiani I. Tillard P.. (2003). Regulation of the nitrate transporter gene AtNRT2. 1 in arabidopsis thaliana: responses to nitrate, amino acids and developmental stage. Plant Mol. Biol. 52 (3), 689–703. doi: 10.1023/a:1024899808018 12956537

[B79] Ninkovic V. Markovic D. Dahlin I. (2016). Decoding neighbour volatiles in preparation for future competition and implications for tritrophic interactions. Perspect. Plant Ecology Evol. Systematics 23, 11–17. doi: 10.1016/j.ppees.2016.09.005

[B80] Nguyen C. (2009). Rhizodeposition of organic c by plant: mechanisms and controls. Sustain. Agric., 97–123. doi: 10.1007/978-90-481-2666-8_9

[B81] Ni J. Yu Z. Du G. Zhang Y. Taylor J. L. Shen C.. (2016). Heterologous expression and functional analysis of rice GLUTAMATE RECEPTOR-LIKE family indicates its role in glutamate triggered calcium flux in rice roots. Rice 9 (1), 1–14. doi: 10.1186/s12284-016-0081-x 26956369 PMC4783324

[B82] Niemeyer H. M. (2009). Hydroxamic acids derived from 2-hydroxy-2 h-1, 4-benzoxazin-3 (4 h)-one: Key defense chemicals of cereals. J. Agric. Food Chem. 57 (5), 1677–1696. doi: 10.1021/jf8034034 19199602

[B83] Nozoye T. Nagasaka S. Kobayashi T. Takahashi M. Sato Y. Sato Y.. (2011). Phytosiderophore efflux transporters are crucial for iron acquisition in graminaceous plants. J. Biol. Chem. 286 (7), 5446–5454. doi: 10.1074/jbc.M110.180026 21156806 PMC3037657

[B84] Nozoye T. Nakanishi H. Nishizawa N. K. (2013). Characterizing the crucial components of iron homeostasis in the maize mutants ys1 and ys3. PloS One 8 (5), e62567. doi: 10.1371/journal.pone.0062567 23667491 PMC3648533

[B85] Olanrewaju O. S. Ayangbenro A. S. Glick B. R. Babalola O. O. (2019). Plant health: feedback effect of root exudates-rhizobiome interactions. Appl. Microbiol. Biotechnol. 103 (3), 1155–1166. doi: 10.1007/s00253-018-9556-6 30570692 PMC6394481

[B86] Ourry M. Lebreton L. Chaminade V. Guillerm-Erckelboudt A. Y. Hervé M. Linglin J.. (2018). Influence of belowground herbivory on the dynamics of root and rhizosphere microbial communities. Front. Ecol. Evol. 6, 91. doi: 10.3389/fevo.2018.00091

[B87] Park Y. S. Ryu C. M. (2021). Understanding plant social networking system: Avoiding deleterious microbiota but calling beneficials. Int. J. Mol. Sci. 22 (7), 3319. doi: 10.3390/ijms22073319 33805032 PMC8037233

[B88] Paterson E. Sim A. Standing D. Dorward M. McDonald A. J. S. (2006). Root exudation from hordeum vulgare in response to localized nitrate supply. J. Exp. Bot. 57 (10), 2413–2420. doi: 10.1093/jxb/erj214 16766600

[B89] Philippot L. Raaijmakers J. M. Lemanceau P. Van Der Putten W. H. (2013). Going back to the roots: The microbial ecology of the rhizosphere. Nat. Rev. Microbiol. 11 (11), 789–799. doi: 10.1038/nrmicro3109 24056930

[B90] Phour M. Sehrawat A. Sindhu S. S. Glick B. R. (2020). Interkingdom signaling in plant-rhizomicrobiome interactions for sustainable agriculture. Microbiol. Res. 241, 126589. doi: 10.1016/j.micres.2020.126589 32927204

[B91] Pieterse C. M. de Jonge R. Berendsen R. L. (2016). The soil-borne supremacy. Trends Plant Sci. 21 (3), 171–173. doi: 10.1016/j.tplants.2016.01.018 26853594

[B92] Pieterse C. M. Zamioudis C. Berendsen R. L. Weller D. M. Van Wees S. C. Bakker P. A. (2014). Induced systemic resistance by beneficial microbes. Annu. Rev. Phytopathol. 52, 347–375. doi: 10.1146/annurev-phyto-082712-102340 24906124

[B93] Pineda A. Zheng S. J. van Loon J. J. Pieterse C. M. Dicke M. (2010). Helping plants to deal with insects: the role of beneficial soil-borne microbes. Trends Plant Sci. 15 (9), 507–514. doi: 10.1016/j.tplants.2010.05.007 20542720

[B94] Preece C. Peñuelas J. (2020). A return to the wild: root exudates and food security. Trends Plant Sci. 25 (1), 14–21. doi: 10.1016/j.tplants.2019.09.010 31648938

[B95] Rasmann S. Köllner T. G. Degenhardt J. Hiltpold I. Toepfer S. Kuhlmann U.. (2005). Recruitment of entomopathogenic nematodes by insect-damaged maize roots. Nature 434 (7034), 732–737. doi: 10.1038/nature03451 15815622

[B96] Rasmann S. Turlings T. C. (2016). Root signals that mediate mutualistic interactions in the rhizosphere. Curr. Opin. Plant Biol. 32, 62–68. doi: 10.1016/j.pbi.2016.06.017 27393937

[B97] Rellán-Álvarez R. Andaluz S. Rodríguez-Celma J. Wohlgemuth G. Zocchi G. Álvarez-Fernández A.. (2010). Changes in the proteomic and metabolic profiles of beta vulgaris root tips in response to iron deficiency and resupply. BMC Plant Biol. 10 (1), 1–15. doi: 10.1186/1471-2229-10-120 20565974 PMC3017792

[B98] Rodríguez-Celma J. Lin W. D. Fu G. M. Abadia J. López-Millán A. F. Schmidt W. (2013). Mutually exclusive alterations in secondary metabolism are critical for the uptake of insoluble iron compounds by arabidopsis and medicago truncatula. Plant Physiol. 162 (3), 1473–1485. doi: 10.1104/pp.113.220426 23735511 PMC3707556

[B99] Rodríguez-Celma J. Vázquez-Reina S. Orduna J. Abadía A. Abadía J. Álvarez-Fernández A.. (2011). Characterization of flavins in roots of fe-deficient strategy i plants, with a focus on medicago truncatula. Plant Cell Physiol. 52 (12), 2173–2189. doi: 10.1093/pcp/pcr149 22039102

[B100] Rolfe S. A. Griffiths J. Ton J. (2019). Crying out for help with root exudates: adaptive mechanisms by which stressed plants assemble health-promoting soil microbiomes. Curr. Opin. Microbiol. 49, 73–82. doi: 10.1016/j.mib.2019.10.003 31731229

[B101] Ross-Elliott T. J. Jensen K. H. Haaning K. S. Wager B. M. Knoblauch J. Howell A. H.. (2017). Phloem unloading in arabidopsis roots is convective and regulated by the phloem-pole pericycle. elife 6, e24125. doi: 10.7554/eLife.24125.022 28230527 PMC5365319

[B102] Rudrappa T. Czymmek K. J. Paré P. W. Bais H. P. (2008). Root-secreted malic acid recruits beneficial soil bacteria. Plant Physiol. 148 (3), 1547–1556. doi: 10.1104/pp.108.127613 18820082 PMC2577262

[B103] Sarita S. Shaikh NB Shaikh N Rochlani A Dalwani A Sharma S. (2022). Rhizobacteria that promote plant growth and their impact on root system architecture, root development, and function. Acta Sci. Microbiol. 5 (4), 53–62. doi: 10.31080/ASMI.2022.05.1035

[B104] Sasse J. Martinoia E. Northen T. (2018). Feed your friends: do plant exudates shape the root microbiome? Trends Plant Sci. 23 (1), 25–41. doi: 10.1016/j.tplants.2017.09.003 29050989

[B105] Savage J. A. Clearwater M. J. Haines D. F. Klein T. Mencuccini M. Sevanto S.. (2016). Allocation, stress tolerance and carbon transport in plants: How does phloem physiology affect plant ecology? Plant Cell Environ. 39 (4), 709–725. doi: 10.1111/pce.12602 26147312

[B106] Schneider T. Keiblinger K. M. Schmid E. Sterflinger-Gleixner K. Ellersdorfer G. Roschitzki B.. (2012). Who is who in litter decomposition? metaproteomics reveals major microbial players and their biogeochemical functions. ISME J. 6 (9), 1749–1762. doi: 10.1038/ismej.2012.11 22402400 PMC3498922

[B107] Schrempf H. Dworkin M. Falkow S. Rosenberg E. Schleifer K. H. Stackebrandt E. (2007). “Biology of streptomycetes,” in The prokaryotes, a handbook on the biology of bacteria, 3rd edn. Eds. Dworkin M. Falkow S. Rosenberg E. Schleifer K. H. Stackebrandt E. (New York, NY: Springer Verlag).

[B108] Schulz-Bohm K. Gerards S. Hundscheid M. Melenhorst J. de Boer W. Garbeva P. (2018). Calling from distance: attraction of soil bacteria by plant root volatiles. ISME J. 12 (5), 1252–1262. doi: 10.1038/s41396-017-0035-3 29358736 PMC5931972

[B109] Sharifi R. Ryu C. M. (2021). Social networking in crop plants: Wired and wireless cross-plant communications. Plant Cell Environ. 44 (4), 1095–1110. doi: 10.1111/pce.13966 33274469 PMC8049059

[B110] Stotz H. U. Sawada Y. Shimada Y. Hirai M. Y. Sasaki E. Krischke M.. (2011). Role of camalexin, indole glucosinolates, and side chain modification of glucosinolate-derived isothiocyanates in defense of arabidopsis against sclerotinia sclerotiorum. Plant J. 67 (1), 81–93. doi: 10.1111/j.1365-313X.2011.04578.x 21418358

[B111] Trivedi P. Batista B. D. Bazany K. E. Singh B. K. (2022). Plant–microbiome interactions under a changing world: Responses, consequences and perspectives. New Phytol 234 (6), 1951–1959. doi: 10.1111/nph.18016 35118660

[B112] Trognitz F. Hackl E. Widhalm S. Sessitsch A. (2016). The role of plant–microbiome interactions in weed establishment and control. FEMS Microbiol. Ecol. 92 (10). doi: 10.1093/femsec/fiw138 27387910

[B113] Ulbrich T. C. Rivas-Ubach A. Tiemann L. K. Friesen M. L. Evans S. E. (2022). Plant root exudates and rhizosphere bacterial communities shift with neighbor context. Soil Biol. Biochem. 172, 108753. doi: 10.1016/j.soilbio.2022.108753

[B114] Uroz S. Buée M. Murat C. Frey-Klett P. Martin F. (2010). Pyrosequencing reveals a contrasted bacterial diversity between oak rhizosphere and surrounding soil. Environ. Microbiol. Rep. 2 (2), 281–288. doi: 10.1111/j.1758-2229.2009.00117.x 23766079

[B115] Usman M. Ho-Plágaro T. Frank H. E. Calvo-Polanco M. Gaillard I. Garcia K.. (2021). Mycorrhizal symbiosis for better adaptation of trees to abiotic stress caused by climate change in temperate and Boreal forests. Front. Forests Global Change 141. doi: 10.3389/ffgc.2021.742392

[B116] van der Heijden M. G. Martin F. M. Selosse M. A. Sanders I. R. (2015). Mycorrhizal ecology and evolution: the past, the present, and the future. New Phytol. 205 (4), 1406–1423. doi: 10.1111/nph.13288 25639293

[B117] Vincill E. D. Bieck A. M. Spalding E. P. (2012). Ca2+ conduction by an amino acid-gated ion channel related to glutamate receptors. Plant Physiol. 159 (1), 40–46. doi: 10.1104/pp.112.197509 22447719 PMC3375973

[B118] Vorwieger A. Gryczka C. Czihal A. Douchkov D. Tiedemann J. Mock H. P.. (2007). Iron assimilation and transcription factor controlled synthesis of riboflavin in plants. Planta 226 (1), 147–158. doi: 10.1007/s00425-006-0476-9 17260143

[B119] Vranova V. Rejsek K. Skene K. R. Janous D. Formanek P. (2013). Methods of collection of plant root exudates in relation to plant metabolism and purpose: A review. J. Plant Nutr. Soil Sci. 176 (2), 175–199. doi: 10.1002/jpln.201000360

[B120] Wang B. Qiu Y. L. (2006). Phylogenetic distribution and evolution of mycorrhizas in land plants. Mycorrhiza 16 (5), 299–363. doi: 10.1007/s00572-005-0033-6 16845554

[B121] Wasternack C. Hause B. (2013). Jasmonates: Biosynthesis, perception, signal transduction and action in plant stress response, growth and development. an update to the 2007 review in annals of botany. Ann. Bot. 111 (6), 1021–1058. doi: 10.1093/aob/mct067 23558912 PMC3662512

[B122] Wen T. Zhao M. Yuan J. Kowalchuk G. A. Shen Q. (2021). Root exudates mediate plant defense against foliar pathogens by recruiting beneficial microbes. Soil Ecol. Lett. 3 (1), 42–51. doi: 10.1007/s42832-020-0057-z

[B123] Weng W. Yan J. Zhou M. Yao X. Gao A. Ma C.. (2022). Roles of arbuscular mycorrhizal fungi as a biocontrol agent in the control of plant diseases. Microorganisms 10 (7), 1266. doi: 10.3390/microorganisms10071266 35888985 PMC9317293

[B124] Wentzell A. M. Kliebenstein D. J. (2008). Genotype, age, tissue, and environment regulate the structural outcome of glucosinolate activation. Plant Physiol. 147 (1), 415–428. doi: 10.1104/pp.107.115279 18359845 PMC2330308

[B125] Xu X. Chen Y. Li B. Zhang Z. Qin G. Chen T.. (2022). Molecular mechanisms underlying multi-level defense responses of horticultural crops to fungal pathogens. Horticulture Res. 9. doi: 10.1093/hr/uhac066 PMC9113409 35591926

[B126] Xu X. Fang P. Zhang H. Chi C. Song L. Xia X.. (2019). Strigolactones positively regulate defense against root-knot nematodes in tomato. J. Exp. Bot. 70 (4), 1325–1337. doi: 10.1093/jxb/ery439 30576511 PMC6382333

[B127] Xu S. Liao C. J. Jaiswal N. Lee S. Yun D. J. Lee S. Y.. (2018). Tomato PEPR1 ORTHOLOG RECEPTOR-LIKE KINASE1 regulates responses to systemin, necrotrophic fungi, and insect herbivory. Plant Cell 30 (9), 2214–2229. doi: 10.1105/tpc.17.00908 30131419 PMC6181013

[B128] Yang H. Zhang Q. Dai Y. Liu Q. Tang J. Bian X.. (2015). Effects of arbuscular mycorrhizal fungi on plant growth depend on root system: a meta-analysis. Plant Soil 389 (1), 361–374. doi: 10.1007/s11104-014-2370-8

[B129] Yuan J. Zhao J. Wen T. Zhao M. Li R. Goossens P.. (2018). Root exudates drive the soil-borne legacy of aboveground pathogen infection. Microbiome 6 (1), 1–12. doi: 10.1186/s40168-018-0537-x 30208962 PMC6136170

[B130] Zhang C. Feng C. Zheng Y. Wang J. Wang F. (2020). Root exudates metabolic profiling suggests distinct defense mechanisms between resistant and susceptible tobacco cultivars against black shank disease. Front. Plant Sci. 11, 559775. doi: 10.3389/fpls.2020.559775 33013978 PMC7511587

[B131] Zhang K. Rengel Z. Zhang F. White P. J. Shen J. (2022). Rhizosphere engineering for sustainable crop production: Entropy-based insights. Trends Plant Sci, S1360-1385(22)00307-7. Advance online publication. doi: 10.1016/j.tplants.2022.11.008 36470795

[B132] Zhao J. Davis L. C. Verpoorte R. (2005). Elicitor signal transduction leading to production of plant secondary metabolites. Biotechnol. Adv. 23 (4), 283–333. doi: 10.1016/j.biotechadv.2005.01.003 15848039

